# Wubi Shanyao pills ameliorate diet-induced postmenopausal osteoporosis in mice by enhancing calcium absorption

**DOI:** 10.3389/fphar.2025.1660284

**Published:** 2025-10-13

**Authors:** Xiaorui Su, Xiaohu Jin, Jingjing Yu, Meiqiu Yan, Jie Su, Guiyuan Lv, Suhong Chen

**Affiliations:** School of Pharmaceutical Sciences, Zhejiang Chinese Medical University, Hangzhou, Zhejiang, China

**Keywords:** calcium absorption, gut-kidney-bone, postmenopausal osteoporosis, TRPV5/6 channels, vitamin D receptor, Wubi Shanyao Pills

## Abstract

**Objective:**

Wubi Shanyao Pills (WSP) is a traditional Chinese botanical formulation known for its gastrointestinal and renal benefits, yet its pharmacological effects on postmenopausal osteoporosis (PMOP) are not well elucidated. This study aimed to evaluate the therapeutic potential of WSP in a diet-induced PMOP model and to investigate its underlying mechanisms related to calcium absorption.

**Methods:**

A PMOP-like model was established in perimenopausal mice using a low-calcium, high-phosphorus diet. The mice were treated daily with WSP (0.375, 0.75, or 1.5 g/kg) or alendronate (ALN) (0.14 g/kg). After 17 weeks of treatment, bone microstructure was assessed via small animal CT, along with evaluation of systemic physiological parameters and hematological profiles. Histopathological examinations of the ileum, kidney, and femur were conducted using hematoxylin-eosin (H&E) staining, Alcian blue-periodic acid-Schiff (AB-PAS), and Masson staining. Serum calcium and phosphorus levels were measured by enzyme-linked immunosorbent assay (ELISA). The expression levels of calcium absorption-related proteins were analyzed using immunohistochemistry (IHC), immunofluorescence (IF), Western blotting, and quantitative real-time polymerase chain reaction (qRT-PCR).

**Results:**

WSP exhibited notable pharmacological effects by improving bone mass/quality and serum calcium/phosphorus levels in diet-induced PMOP mice, mediated via upregulating key calcium transport proteins: transient receptor potential vanilloid 5 (TRPV5) and calcium-binding protein (CABP) in the kidney, transient receptor potential vanilloid 6 (TRPV6) and CABP in the ileum, and vitamin D receptor (VDR) in the femur; moreover, WSP reversed PMOP-associated anemia and facilitated tissue structural repair in the kidney, ileum, and femur.

**Conclusion:**

WSP modulates diet-induced PMOP pathology by promoting calcium absorption via the restoration of organ integrity and regulation of the TRPV5/TRPV6–CABP and VDR-mediated calcium metabolism pathways, thereby underlying its pharmacological effects.

## 1 Introduction

Postmenopausal osteoporosis (PMOP) is a systemic metabolic bone disorder characterized by a reduction in bone mass per unit volume, deterioration of bone microstructure, decreased bone mineral density (BMD), and diminished bone strength. These pathological changes lead to increased bone fragility and a higher risk of fractures, primarily due to estrogen deficiency following menopause. PMOP is associated with accelerated bone turnover, in which bone resorption exceeds bone formation, resulting in rapid bone loss (Wang C. et al., 2024). This condition predominantly affects trabecular bone, rendering the vertebrae and long bones particularly susceptible to fractures. The pathogenesis of osteoporosis is multifactorial, involving factors such as aging, endocrine disturbances, and calcium homeostasis disruption, as well as influences from immune function, nutrition, and genetic factors (Wang Y. et al., 2024). In 2021, a total of 219,552 postmenopausal women died due to low BMD, which includes osteopenia and osteoporosis. Over a 31-year period, the number of such deaths has doubled, with an average annual increase of approximately 2.3% (Liang et al., 2025). Although bone loss initiates as early as 2–3 years before menopause, it peaks between 1 year before and 2 years after the final menstrual period (Lizneva et al., 2018). The mortality rate caused by PMOP is 15.17 times higher than that in premenopausal women, underscoring the postmenopausal period as a critical window of risk for metabolic bone diseases (Liang et al., 2025). As China transitions into an aging society, the incidence of osteoporosis has been steadily rising, with a prevalence of 37.7% among individuals aged 60 and older. This condition disproportionately affects women, emerging as a significant public health concern, ranking just after cardiovascular diseases, cerebrovascular diseases, hypertension, and diabetes (Quah et al., 2022; Xiao et al., 2022). By 2045, global deaths and disability attributable to PMOP among elderly women are projected to increase further, highlighting a growing worldwide demand for improved bone health management (Liang et al., 2025).

Minerals are essential for maintaining human health and play pivotal roles in numerous physiological processes. Among these, calcium is the most abundant mineral in the human body, second only to oxygen, carbon, hydrogen, and nitrogen. An adult human body typically contains between 1,200 and 2,400 g of calcium, accounting for 1.5%–2.0% of total body weight (Gordan and Vaughan, 1986). Calcium is involved in nearly every vital process, including the maintenance of bone health, cellular metabolism, enzyme regulation, muscle contraction, heart function, nerve transmission, and blood coagulation (Bass and Chan, 2006). Its primary function is to form and support the structure of bones and teeth, with bone tissue storing 99% of the body’s calcium in the form of hydroxyapatite (Deluque et al., 2025). In addition to its structural roles, adequate calcium intake is crucial for maintaining bone health, preventing osteoporosis and dental caries, and reducing bone loss (Marino et al., 2023). The kidneys, small intestine, and bones play pivotal roles in maintaining the body’s calcium homeostasis. Under normal conditions, these organs work in concert to regulate blood calcium levels, ensuring dynamic equilibrium (Papadopoulou et al., 2021). Dietary calcium serves as the primary source of calcium for the body, making the absorption of calcium in the small intestine crucial for maintaining calcium balance (Lieben et al., 2012). The small intestine, located in the abdomen, connects to the large intestine at its distal end, facilitating further digestion and absorption of food. The proximal end of the small intestine, the duodenum, is connected to the stomach through the pylorus. Additionally, the intestinal mucosal epithelium is the primary site of calcium (Ca^2+^) absorption, a critical mineral for bone health (Larussa et al., 2017). Intestinal dysfunction, which may arise from intense external stimuli, impairs the ability of the stomach and intestines to absorb calcium effectively. This results in impaired calcium synthesis in the blood and a diminished capacity to convert and absorb calcium from dietary sources, which can disrupt bone metabolism. Research indicates that individuals with Crohn’s disease (CD) and osteoporosis are more likely to experience chronic gastrointestinal disorders, highlighting the close relationship between the onset of osteoporosis and gastrointestinal dysfunction (Zhong et al., 2022). A 4-year, randomized, double-blind, placebo-controlled study involving 930 healthy older participants with an average age of 61 demonstrated that calcium supplementation is an effective strategy for preventing osteoporosis and fractures in individuals with senile osteoporosis (Bischoff-Ferrari et al., 2008). Consequently, preventing osteoporosis relies heavily on either enhancing the body’s calcium absorption capacity or ensuring adequate calcium intake. Research has shown that colostrum basic protein (CBP) modulates intestinal calcium transporters and calcium homeostasis, enhances bone calcium deposition, and supports bone growth via a tripartite regulatory mechanism encompassing “ileum-dominated paracellular calcium absorption, jejunal transcellular transport, and hormonal regulation of calcium homeostasis.” These findings designate CBP as a novel natural protein target for the prevention and therapy of osteoporosis (Zhang et al., 2024). Additionally, studies have investigated the synergistic application of probiotics and isoflavones in the treatment of PMOP. This method has demonstrated the ability to augment calcium absorption by increasing the expression of intestinal calcium transporters—TRPV6, CABP, and PMCA1b—thus mitigating bone loss and enhancing systemic calcium equilibrium and skeletal health (Harahap et al., 2024).

Currently, the pharmacological management of PMOP includes various agents, such as essential mineral supplements, bone resorption inhibitors, bone formation stimulators, and drugs with novel mechanisms of action. Nonetheless, each therapeutic option presents specific limitations (An et al., 2020). For instance, bisphosphonates, which inhibit osteoclast-mediated bone resorption, are currently first-line therapy for osteoporosis. However, both bisphosphonates and denosumab have been associated with adverse effects such as osteonecrosis of the jaw and atypical femoral fractures (Gehrke et al., 2023). Although cathepsin K inhibitors can reduce the risk of fractures, they are linked to an increased risk of cardiovascular events. Moreover, studies have indicated that oral calcitonin does not significantly reduce fracture incidence. While traditional hormone replacement therapy is effective in alleviating menopausal bone loss, its clinical use is limited by side effects, prompting increased interest in more natural treatment alternatives with improved safety profiles (Chen et al., 2021; Shen et al., 2022). Traditional Chinese Medicine (TCM) has shown considerable potential in the treatment of PMOP, with both compound formulations and single herbal extracts exhibiting scientifically validated efficacy (Wang L. et al., 2024). Despite numerous studies have reported on the use of Chinese patent medicines for PMOP, and several TCM formulas currently employed in clinical practice, most previous investigations have primarily focused on clinical outcomes rather than systematically elucidating the underlying molecular mechanisms or multi-organ crosstalk involved in bone metabolism (Liu et al., 2025). WSP is a classic Chinese patent medicine with a history dating back to the Tang Dynasty. It is currently approved for sale in the Chinese market and is manufactured by Hangzhou Hu Qing Yu Tang Pharmaceutical Co., Ltd. Composed of 12 botanical drugs, this preparation can alleviate renal tissue damage in aged mice, improve renal function, and effectively delay reproductive aging (Xiaohu et al., 2024).

Pharmacological research demonstrates that the principal chemical components of *Rehmannia glutinosa (Gaertn.)* Libosch. ex DC. exhibit notable therapeutic effects, such as osteoporosis prevention, anti-aging properties, anti-thrombotic activity, support for recovery from bone marrow suppression, and immune function modulation (Lim and Kim, 2013; Zhou et al., 2015). *Dioscorea oppositifolia* L., a prominent medicinal and culinary plant, is essential for tonifying the spleen, nourishing the stomach, and enhancing fluid production, notably concerning pulmonary health. The immunomodulatory impact of *Dioscorea oppositifolia* L. on the intestinal tract is chiefly evidenced by its capacity to restore the intestinal mucosa. This repair improves the mucosal protective function of the intestinal wall, consequently modulating immunological responses and allowing the intestine to efficiently execute its immune activities (Guo et al., 2024; Xue et al., 2019). *Cuscuta australis* var. *australis* exhibits anticancer, antioxidant, and endocrine-immune regulating characteristics (Moon et al., 2019). The extract of *C. australis* var. *australis*, as indicated by Liu et al. (2020), possesses therapeutic advantages in osteoporosis and can promote osteoblast proliferation. A possible mechanism for these effects includes suppressing osteoclast differentiation and alleviating estrogen deficiency, which contributes to bone loss; this pathway is also linked to the anti-osteoporotic properties of *C. australis* var. *australis* and its bioactive constituents (Wang et al., 2023). We anticipate that the synergistic effects of these botanical medications may effectively alleviate the symptoms of osteoporosis, based on their pharmacological qualities.

TCMs that tonify the kidney play a crucial role in promoting bone formation, inhibiting bone resorption, stimulating sex hormone secretion, suppressing oxidative stress, and regulating calcium-phosphorus metabolism. According to the TCM theory of “the kidney governing bone,” kidney-tonifying herbs are often employed in the treatment of PMOP. Additionally, TCM etiology identifies “deficiency of both the spleen and kidney” as a key pathological mechanism underlying osteoporosis. As a well-known kidney-tonifying formula, WSP has been a focus of our prior research. In prior studies, we found that WSP had a significant restorative impact on intestinal function in animal models of “Folium senna-induced diarrhea” and “dietary disorders” combined with “overwork.” Building on these findings, the present study utilized 13-month-old female mice, which were fed a low-calcium, high-phosphorus diet to better replicate the clinical features of menopausal osteoporosis. We systematically evaluated the physical manifestations of the disease, changes in bone mass and mineral content, and the expression of calcium absorption mechanisms in the kidneys, intestines, and bones. This comprehensive approach was used to investigate the effects and underlying mechanisms of WSP in the context of diet-induced PMOP mice. By examining these multiple aspects, we aimed to elucidate how WSP may modulate calcium metabolism and bone health, giving us valuable knowledge about its potential therapeutic role in the management of PMOP.

## 2 Materials and methods

### 2.1 Chemicals and reagents

WSP (0.075 g/pill, 9 g/bag, 2313904) was provided by Hangzhou Hu Qing Yu Tang Pharmaceutical Co., Ltd. This formulation consisted of 12 botanical drugs, including *Dioscorea oppositifolia* L. (Shanyao in Chinese), *Eucommia ulmoides* Oliv. (Duzhong, in Chinese), *Rehmannia glutinosa* (Gaertn.) *Libosch*. ex DC. (Shudihuang in Chinese), *Cuscuta australis* var. *australis* (Tusizi in Chinese), *Cornus officinalis* Siebold & Zucc. (Shanzhuyu in Chinese), *Poria cocos* (Schw.) Wolf (Fuling in Chinese), *Morinda officinalis* F.C.How (Bajitian in Chinese), *Alisma plantago-aquatica* L. (Zexie in Chinese), *Achyranthes bidentata* Blume (Niuxi in Chinese), *Kadsura coccinea* (Lem.) A.C.Sm. (Wuweizi in Chinese), *Cistanche deserticola* Ma (Roucongrong in Chinese), and *Halloysitum Rubrum* (Chishizhi in Chinese). The aforementioned plants have been verified with reference to http://mpns.kew.org/mpns-portal/. Fungal names had been verified with reference to Chinese Pharmacopoeia 2020. ALN (70 mg/tablet, 22211014) were supplied by Beijing Fuyuan Pharmaceutical Co., Ltd. Calcium (Ca, 20240406) and Phosphorus (P, 20240321) were obtained from Nanjing Jiancheng Bioengineering Institute. Parathyroid hormone (PTH, 202404), Calcitonin (CT, 202405), and 1,25-dihydroxyvitamin D_3_ (1,25-(OH)_2_D_3_, 202408) were provided by Jiangsu Enzyme Immunoassay Industrial Co., Ltd. Estradiol (E_2_, RXJ203008M) was supplied by Quanzhou Ruixin Biotechnology Co., Ltd. VDR (YM4683) and CABP (YN5655) antibodies were purchased from Immunoway Biotechnology Company (North America). TRPV6 (13411-1-AP) antibody was obtained from Proteintech Group, Inc. (Wuhan, China). GAPDH (ET1601-4) and beta Actin (HA722023) antibodies were purchased from Hangzhou Huaan Biotechnology Co., Ltd. TRPV5 (ab137028) antibody was obtained from Abcam PLC (United Kingdom).

### 2.2 Animals and study design

Seventy 10-month-old female ICR mice (SPF grade) were purchased from Shanghai Bikai Keyi Biotechnology Co., Ltd., with the certificate number 20230009002909 and animal production license number SCXK (Hu) 2023-0009. After 3 months of standard housing, 50 perimenopausal mice were selected based on continuous observation of vaginal smears for 10 days. The selected mice were randomly divided into five groups: model group (MC, n = 10), ALN group (0.14 mg/kg), WSP low-dosage group (WSP-L 0.375 g/kg), WSP middle-dosage group (WSP-M 0.75 g/kg), and WSP high-dosage group (WSP-H 1.5 g/kg), with 10 mice in each group. All groups were administered a “low calcium, high phosphorus” diet (provided by Kuibu Qianli Biotechnology Co., Ltd.), containing 0.1% calcium and 1.2% phosphorus. In addition, 10 female ICR mice of specific pathogen-free (SPF) grade aged 6–8 weeks were purchased from Hangzhou Qizhen Laboratory Animal Technology Co., Ltd., with the certificate number 20240328Abzz0100999654 and animal production license number SCXK (Zhe) 2022-0005, and served as the normal control group (NC), receiving a standard diet. The treatment groups received daily oral gavage of the drug at a body weight-adjusted dose for 17 consecutive weeks, whereas the normal control group received an equivalent volume of normal saline administered on the same schedule. The animal study was approved by the Animal Protection and Welfare Committee of Zhejiang Chinese Medical University (IACUC-202402-16) in February 2024. All efforts were made to minimize animal suffering during surgical procedures.

### 2.3 Determination of WSP content

A precise amount of WSP was finely ground and accurately weighed (2 g). It was then added to 25 mL of 50% methanol, followed by ultrasonic treatment (250 W, 40 kHz) for 30 min. After cooling, the weight was remeasured, and 50% methanol was added to compensate for the weight loss. The mixture was shaken well and filtered, and the resulting filtrate was collected for subsequent HPLC analysis. The quantitative analysis of echinacoside (C_35_H_46_O_20_), verbascoside (C_29_H_36_O_15_), and schisandrin (C_24_H_32_O_7_) in WSP was conducted in accordance with the guidelines stipulated in the Chinese Pharmacopoeia (2015 edition, General Principle 0512) utilizing an Agilent 1200 HPLC system (DE62962036). Chromatographic separation was achieved on an Ultimate LP-C18 column (4.6 × 250 mm, 5 μm; serial no.: 011101551) maintained at 25 °C. The mobile phase consisted of (A) acetonitrile and (B) 0.3% phosphoric acid solution, eluted at a flow rate of 1.0 mL/min with the following gradient program: 10%–20% A from 0 to 40 min; 20%–55% A from 40 to 45 min; and held at 55% A from 45 to 60 min. Detection was performed at a wavelength of 254 nm with an injection volume of 10 μL.

### 2.4 Behavioral index detection

After 17 weeks of treatment with the low calcium, high phosphorus diet, grip strength and spontaneous activity frequency of the mice were measured using a YLS-13 A-type grip strength meter and a YLS-1A-type small animal activity monitor, respectively. Rectal temperature was measured using a thermometer, and pain threshold was assessed using a YLS-12A-type tail light nociception meter. Open field tests were conducted to assess the mice’s mental state. Subsequently, blood samples were collected from the orbital vein and placed into EDTA-containing anticoagulant tubes for analysis of various blood cell counts and proportions using a SYSMEX automated hematology analyzer (Japan).

### 2.5 Enzyme-linked immunosorbent assays (ELISA)

During the treatment period, blood samples were also taken from the orbital vein at regular intervals, and serum was separated by centrifugation. Levels of calcium, phosphorus, PTH, CT, 1,25-(OH)_2_D_3_, and E_2_ in the serum were measured using ELISA kits.

### 2.6 Bone CT scan

After 17 weeks of WSP administration, the mice were euthanized via orbital blood collection, and the left femurs were harvested for CT scanning using a small animal micro-CT system.

### 2.7 Histological evaluations (H&E) staining

After euthanasia, femurs, kidneys, and ilea were harvested and fixed in formalin for 72 h. The tissues were then dehydrated, paraffin-embedded, and sectioned into 4 µm thick slices. H&E staining was performed, and histological changes in the femur, kidney, and ileum of the mice from each group were observed under a microscope.

### 2.8 Immunohistochemistry and immunofluorescence

For immunohistochemistry (IHC), the paraffin sections of kidney and femur were dewaxed and incubated with primary antibodies targeting TRPV5 and VDR proteins. Additionally, immunofluorescence (IF) staining was performed. After overnight incubation with CABP, TRPV6, TRPV5, and VDR antibodies, FITC-conjugated AffiniPure Goat Anti-Rabbit IgG (H + L) was added. Samples were subsequently sealed with an anti-fade reagent containing DAPI, and images were captured using a laser confocal microscope (Zeiss SteREO Discovery. V20, Germany).

### 2.9 Western blotting

For protein extraction, kidney, ileum, and femur tissues were placed into grinding tubes with grinding beads and a strong RIPA lysis buffer. The tissues were homogenized using a tissue homogenizer at 60 Hz for 120 s, repeated five times until complete tissue lysis was achieved. The samples were then centrifuged to collect the supernatant, and protein concentrations were measured. Proteins were separated by SDS-PAGE and transferred to Immobilon-P PVDF membranes according to standard protocols. After blocking with rapid blocking buffer, the membranes were incubated overnight at 4 C with primary antibodies against CABP (1:1000), TRPV5 (1:10,000), TRPV6 (1:1000), and VDR (1:1000). On the following day, the membranes were washed with PBST and incubated with secondary antibodies for 2 h. Protein bands were detected using enhanced chemiluminescence (ECL) reagents and visualized with a chemiluminescence imaging system. The resulting images were analyzed using ImageJ software (National Institutes of Health, Bethesda, USA).

### 2.10 Statistics

Statistical analysis was performed using SPSS 26.0 software. Data are presented as mean ± standard deviation ( $\bar{x}\pm s$ ). For pairwise comparisons between two groups, the Wilcoxon rank-sum test was used. For comparisons involving multiple groups, one-way analysis of variance (ANOVA) was performed when the data met the assumptions of normality and homogeneity of variance, followed by Tukey’s Honestly Significant Difference (HSD) test for *post hoc* analysis. In cases where parametric assumptions were violated, the Kruskal–Wallis rank-sum test was applied, followed by Dunn’s test with Bonferroni correction for *post hoc* pairwise comparisons. A P-value of <0.05 was considered statistically significant. Graphs and figures were generated using GraphPad Prism 8.

## 3 Results

### 3.1 Component content of WSP

**FIGURE 1:**
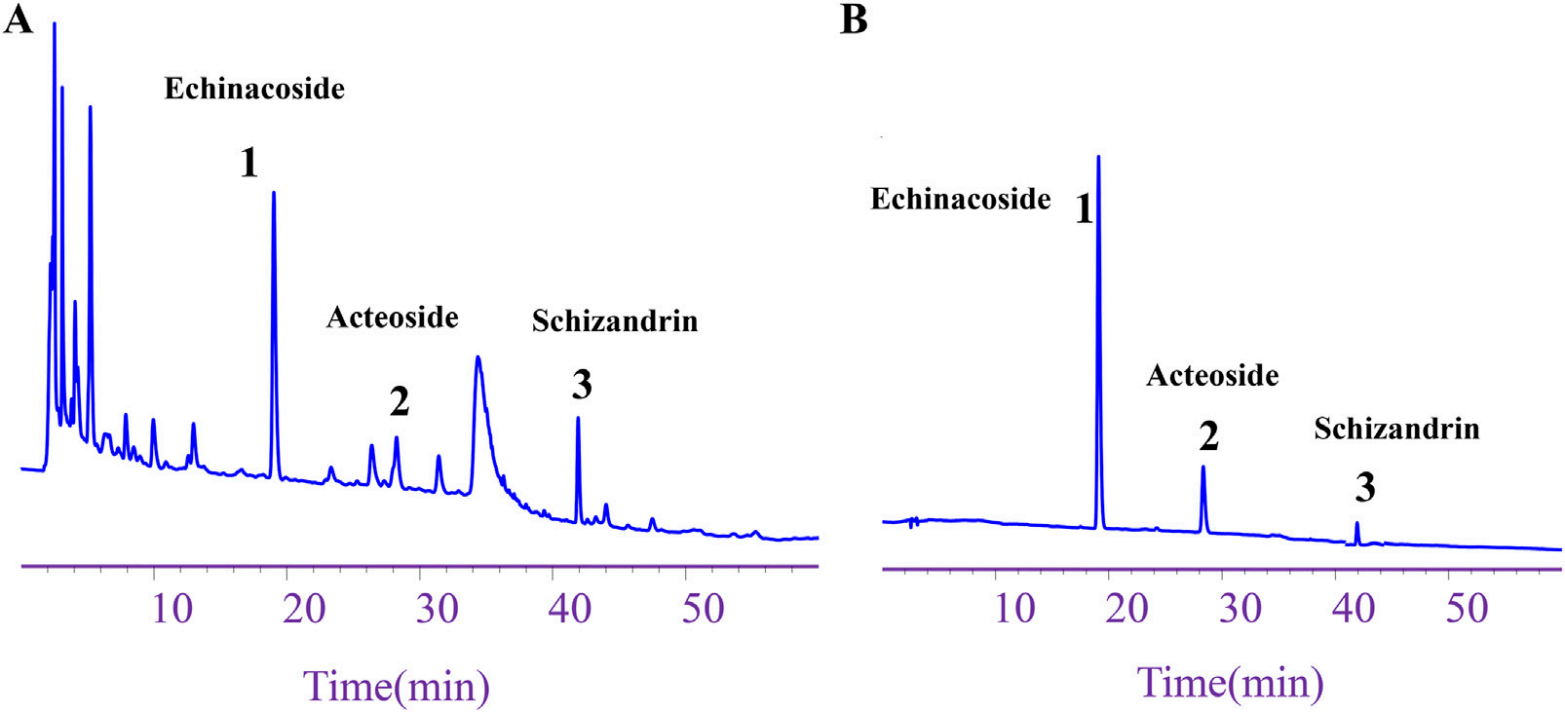
Component content of WSP. **(A)** HPLC chromatogram depicting echinacoside, acteoside, and schizandrin in WSP. **(B)** HPLC chromatograms of the standards for schizandrin, acteoside, and echinacoside in WSP.

The HPLC analysis indicated that the contents of echinacoside, acteoside, and schizandrin in WSP were 2.93 mg/g, 0.69 mg/g, and 0.42 mg/g, respectively. These findings meet the quality standards set by the Chinese Pharmacopoeia, which stipulates that the total amount of echinacoside and acteoside per Gram of WSP must not be less than 2.5 mg, while the content of schizandrin should not be less than 0.1 mg (Figures 1A,B). The results obtained from HPLC confirm the reliable quality of WSP.

### 3.2 WSP improves behavioral performance in diet-induced PMOP mice

**FIGURE 2:**
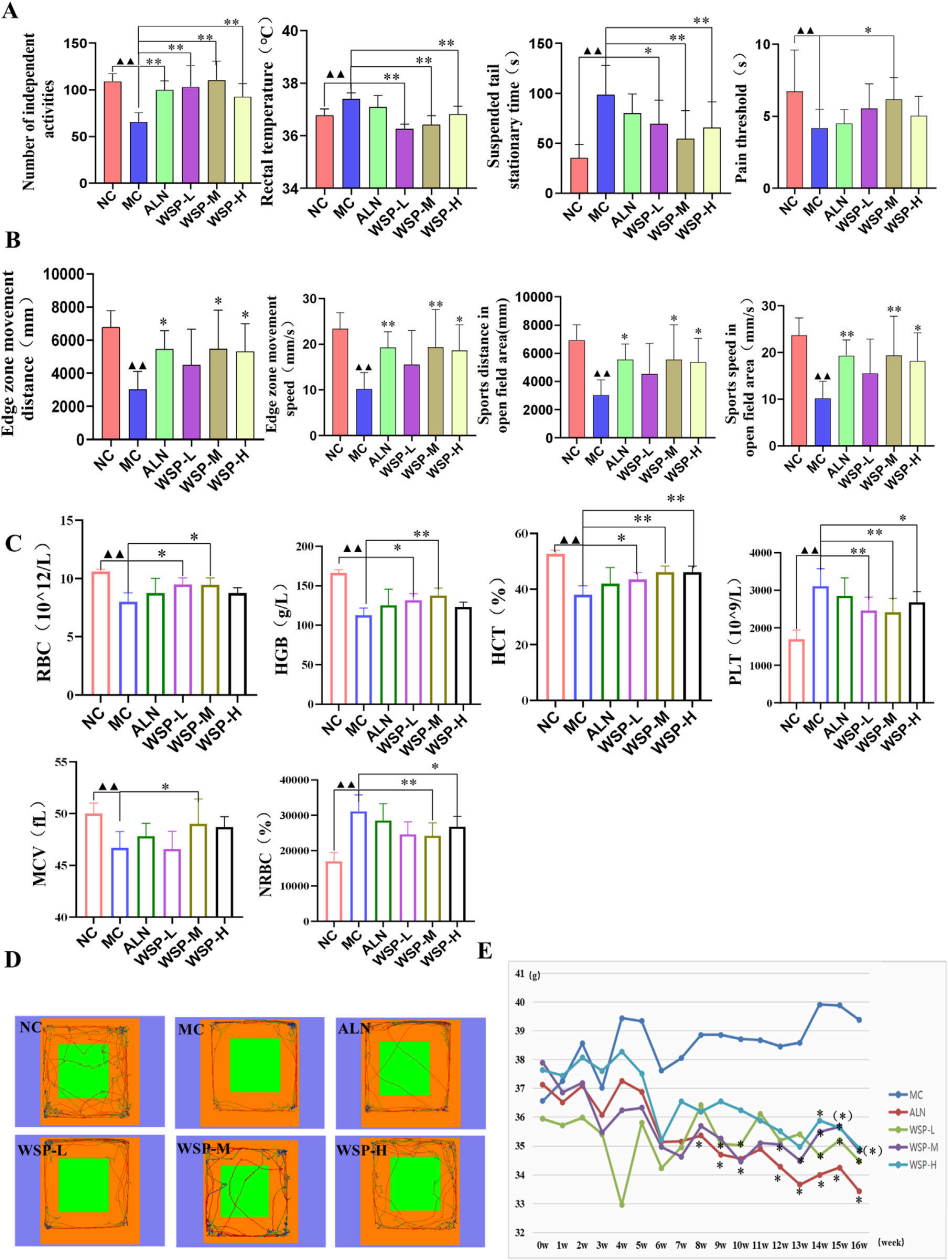
WSP improves the perimenopausal symptoms in diet-induced PMOP mice. **(A)** Number of voluntary activities, anal temperature, tail suspension resting time, pain threshold, n = 10; **(B)** Movement speed and movement time of fringe area and absentia area in absentia detection, n = 6; **(C)** Hematological indexes of RBC, HGB, HCT, PLT, MCV, and NRBC, n = 7–8; **(D)** Absentia trajectory graphs; **(E)** Body weight change of mice in each group. Data are presented as $\bar{x}$ ±s, ^▲▲^P < 0.01, ^▲^P < 0.05, compared with NC, **P < 0.01, *P < 0.05, compared with MC.

Perimenopausal women commonly experience a range of physical, mental, and psychological symptoms, including hot flashes, dizziness, knee and lumbar pain, memory loss, anxiety, and depression. In a murine model of these somatic symptoms, we observed that the model group exhibited significantly higher anal temperatures (P < 0.01) and fewer voluntary activities (P < 0.01) compared to controls (Figure 2A). In terms of mental state, MC mice demonstrated a lower pain threshold (P < 0.01) and a prolonged tail-hanging resting period (P < 0.01) (Figure 2A). Furthermore, MC mice showed an increasing trend in body weight (Figure 2E) and significantly shorter movement distances in both the limbic and absconding zones (P < 0.01), with decreased movement speed in the absconding experiments (P < 0.01) (Figures 2B,D). Routine blood analysis revealed that MC mice exhibited symptoms of anemia, characterized by significantly lower blood levels of red blood cell (RBC), hemoglobin (HGB), hematocrit (HCT), and mean corpuscular volume (MCV) (P < 0.01), while the levels of platelet (PLT) and nucleated red blood cells (NRBC) were significantly elevated (P < 0.01) (Figure 2C). Following treatment with WSP, improvements were observed in both the somatic and mental states of the PMOP mice. Mice in all three WSP dosage groups displayed significantly increased voluntary activity (P < 0.01), lower anal temperatures, and reduced tail-hanging resting times (P < 0.01, 0.05). Additionally, the WSP-M group exhibited higher pain thresholds (P < 0.05). In the limbic and open-field areas, both the distance traveled and movement speed were significantly higher in the WSP-M and WSP-H groups (P < 0.01, 0.05). Body weight in the model mice decreased following WSP administration, with a significant difference observed at week 13 (P < 0.05). WSP treatment also resulted in a reduction of PLT and NRBC levels (P < 0.01, 0.05), while alleviating anemia symptoms and increasing RBC, HGB, HCT, and MCV levels (P < 0.01, 0.05). These results collectively suggest that WSP significantly reduces anxiety-like behaviors, improves exercise capacity, boosts metabolism, decreases body weight, and mitigates anemia, all of which contribute to ameliorating the physical and psychological symptoms associated with PMOP. This indicates that WSP holds potential as a therapeutic agent for addressing the multifaceted symptoms of PMOP.

### 3.3 WSP ameliorates bone microstructure damage in diet-induced PMOP mice

**FIGURE 3:**
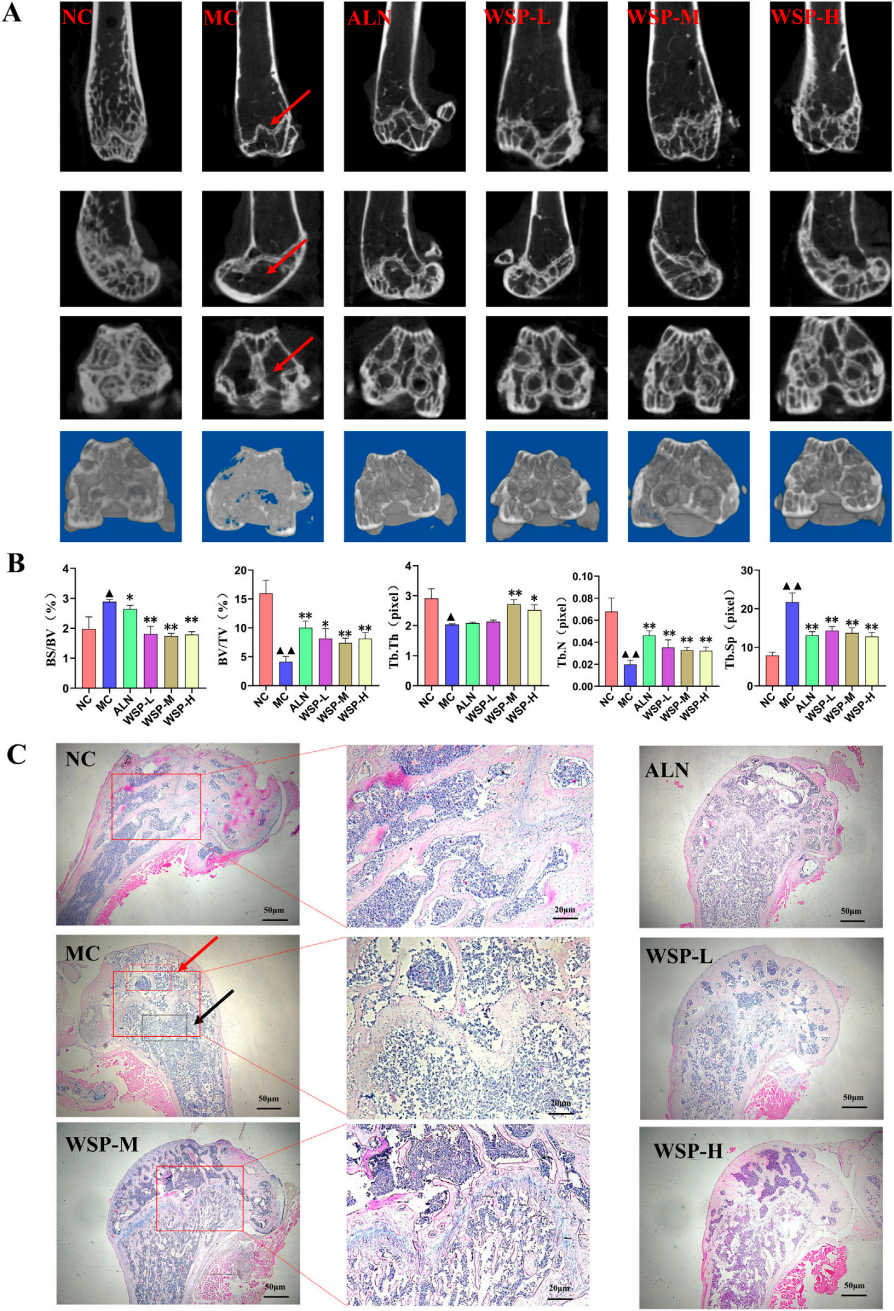
WSP improves bone microstructure in diet-induced PMOP mice. **(A,B)** Bone CT scans of mouse femur (BV/TV, BS/BV, Tb.Th, Tb.N, Tb.Sp); **(C)** H&E staining of mouse femur (The red arrow denotes the damaged areas with sparse and disorganized trabecular bone in diet-induced PMOP Model group (MC); The black arrow indicates the typical pathological sites of trabecular bone fracture and expanded bone marrow cavity in the MC group); Data are presented as $\bar{x}$ ±s, ^▲▲^ *P* < 0.01, ^▲^ *P* < 0.05, compared with NC, **P < 0.01, *P < 0.05, compared with MC.

Bone CT analysis revealed that MC mice exhibited significantly lower bone volume fraction (BV/TV) (P < 0.01), trabecular thickness (Tb.Th) (P < 0.05), and trabecular number (Tb.N) (P < 0.01), while displaying higher bone surface area to volume ratios (BS/BV) (P < 0.05) and trabecular separation (Tb.Sp) (P < 0.01) compared to NC mice. After WSP treatment, femoral BV/TV, Tb.Th, and Tb.N levels significantly increased (P < 0.01, 0.05), while BS/BV and Tb.Sp levels significantly decreased (P < 0.01, 0.05) (Figures 3A,B).

Histological examination using H&E staining showed that the femoral cortical bone plate in the MC group was thinner, more porous, and enlarged, while the cancellous bone trabeculae were thinner, fragmented, and destroyed compared to NC mice. In contrast, WSP-treated groups exhibited notable improvements in bone microstructure and reduced bone damage (Figure 3C). These findings suggest that WSP effectively ameliorates femoral bone damage, increasing bone mass and volume in diet-induced PMOP mice.

### 3.4 WSP regulates calcium and phosphorus levels in diet-induced PMOP mice

**FIGURE 4:**
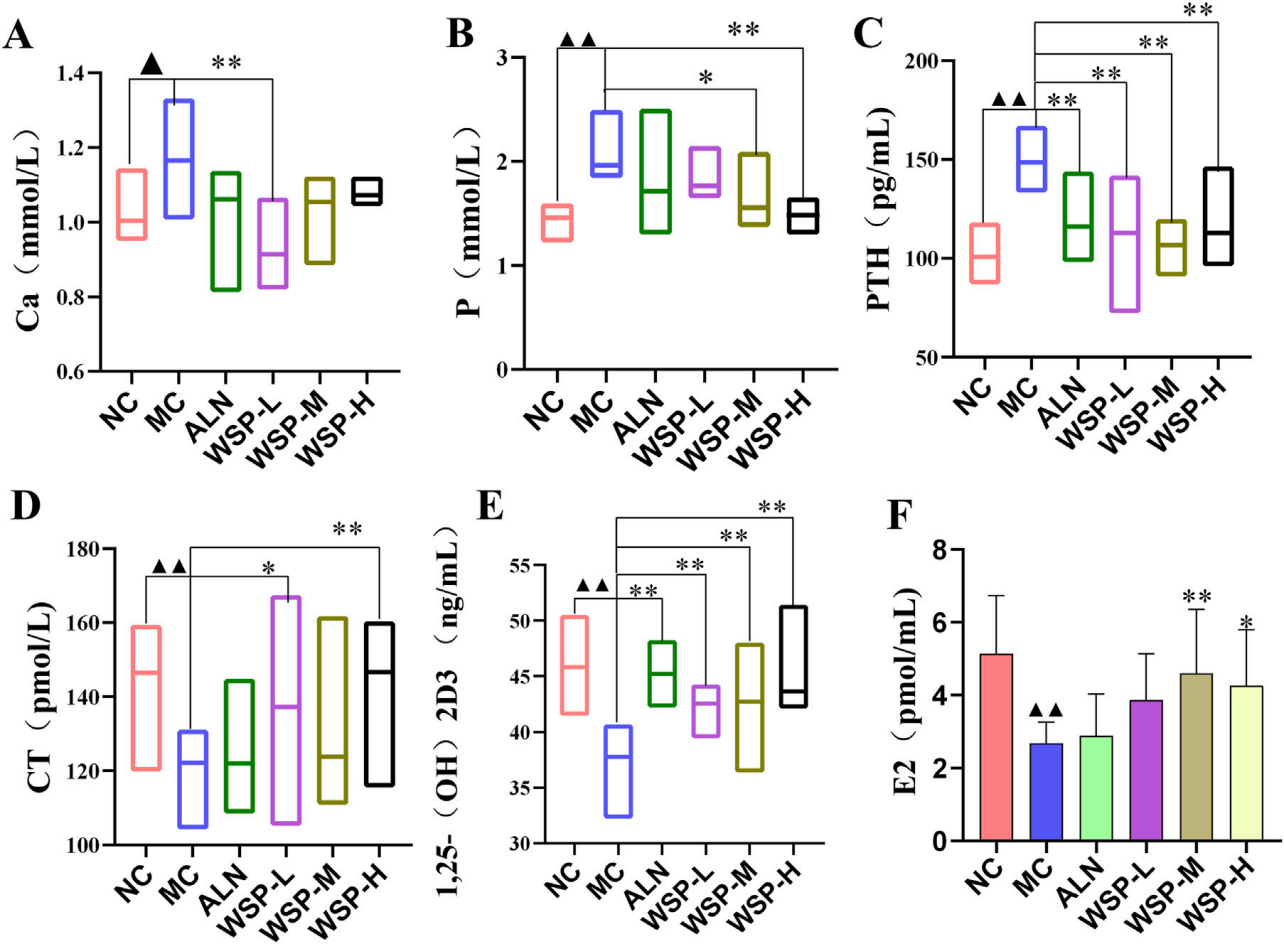
WSP improves *in vivo* calcium and phosphorus levels in diet-induced PMOP mice. **(A)** Ca; **(B)** P; **(C)** PTH; **(D)** CT; **(E)** 1,25-(OH)_2_D_3_; **(F)** E_2_. Data are presented as $\bar{x}$ ±s, ^▲▲^ *P* < 0.01, ^▲^ *P* < 0.05, compared with NC, **P < 0.01, *P < 0.05, compared with MC.

Disorders of calcium metabolism are a key contributor to PMOP. Serum calcium (Ca), phosphorus (P), and PTH levels serve as indirect indicators of bone metabolism. In our study, we observed an increasing trend in serum Ca (P < 0.05), P (P < 0.01), and PTH levels (P < 0.01) in diet-induced PMOP mice (Figures 4A–C), while the levels of CT, 1,25(OH)_2_D_3_, and E_2_ were significantly decreased (P < 0.01) (Figures 4D–F). These changes were effectively reversed following WSP administration (P < 0.01, 0.05). These findings suggest that WSP can effectively increase E_2_ levels, regulate calcium and phosphorus metabolism, and alleviate the symptoms associated with PMOP.

### 3.5 WSP increases intestinal calcium absorption in diet-induced PMOP mice

**FIGURE 5:**
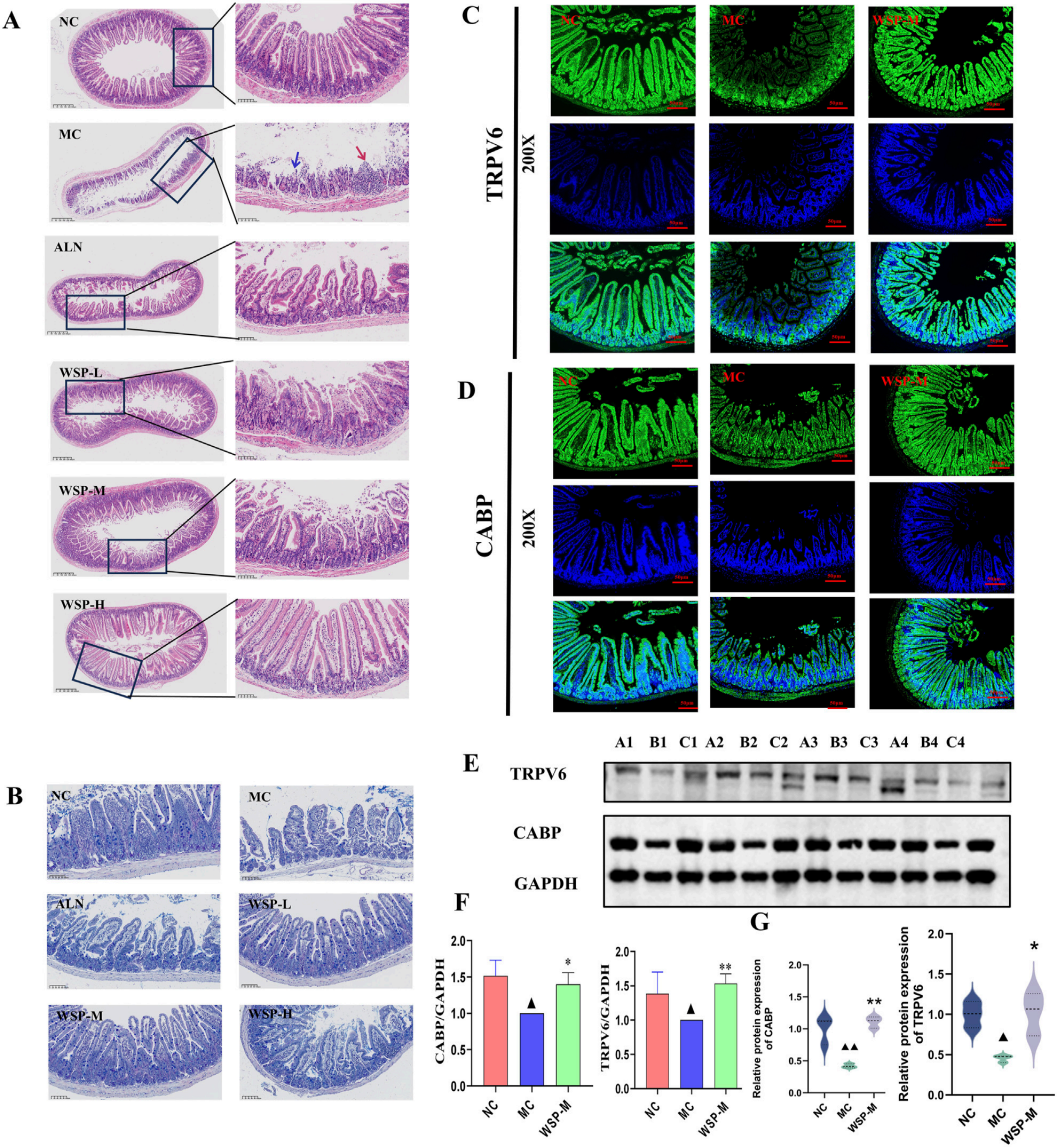
WSP increases intestinal calcium absorption in diet-induced PMOP mice. **(A)** H&E staining of mouse ileum (50x, 400x) (The blue arrow indicates the area with epithelial injury or desquamation; the red arrow indicates the area with epithelial hyperplasia or inflammatory infiltration); **(B)** AB-PAS staining of mouse ileum; **(C,D)** Immunofluorescence to detect the expression of TRPV6, CABP in mouse colon; **(E,F)** Western blot to detect the expression of TRPV6, CABP in ileum tissues (A1∼A4 for NC. B1∼B4 for MC, C1∼C4 for WSP-M group, n = 4). **(G)** Immunofluorescence-based relative quantification of TRPV6 and CABP expression in the intestine. Data are presented as $\bar{x}$ ±s, ^▲▲^ *P* < 0.01, ^▲^ *P* < 0.05, compared with NC, **P < 0.01, *P < 0.05, compared with MC.

Calcium from dietary intake is the sole source of calcium for the body, making calcium absorption in the small intestine crucial for maintaining calcium homeostasis (Lieben et al., 2012). One of the hallmarks of PMOP is increased bone resorption, leading to the release of calcium and phosphorus from bone into the bloodstream and resulting in disrupted calcium homeostasis. This disruption is often accompanied by severe dysfunction of intestinal calcium absorption (Gallagher et al., 1979). H&E staining revealed that the ileal mucosal epithelium of NC mice was structurally intact, with well-organized and tightly packed glands. The villi appeared regular and densely packed, with a high columnar shape, reflecting normal intestinal architecture and calcium absorption capacity; In the ileum of MC mice, the glands were atrophied and irregularly arranged, while the villi were shortened, fragmented, and fused, accompanied by significant infiltration of inflammatory cells. In contrast, the WSP-treated group showed intact mucosal epithelium, with glands neatly arranged and villi densely raised in a regular, orderly fashion, and no signs of inflammatory cell infiltration (Figure 5A). AB-PAS staining was utilized to examine the goblet cells within the intestinal tissues of the mice. The results indicated a significant reduction in the number of goblet cells in the ileum of MC mice compared to NC mice, whereas WSP administration significantly increased the number of goblet cells in the ileum of the treated groups, relative to MC mice (Figure 5B). In immunofluorescence assays, we observed that the expression of calcium channel proteins TRPV6 and CABP in the ileum of MC was significantly lower than that in NC and WSP-M groups (Figures 5C,D). Western blot analysis further confirmed that the protein expression of TRPV6 and CABP in the ileum of MC mice was significantly reduced (P < 0.05), whereas the WSP-M group showed a significant increase in TRPV6 and CABP expression compared to MC mice (P < 0.01, 0.05) (Figures 5E,F). Immunofluorescence quantification further confirmed the results shown in Figures C–F (Figure 5G) (P < 0.01, 0.05). These results suggest that WSP has the potential to ameliorate intestinal damage and enhance calcium absorption.

### 3.6 WSP increases renal calcium uptake in diet-induced PMOP mice

**FIGURE 6:**
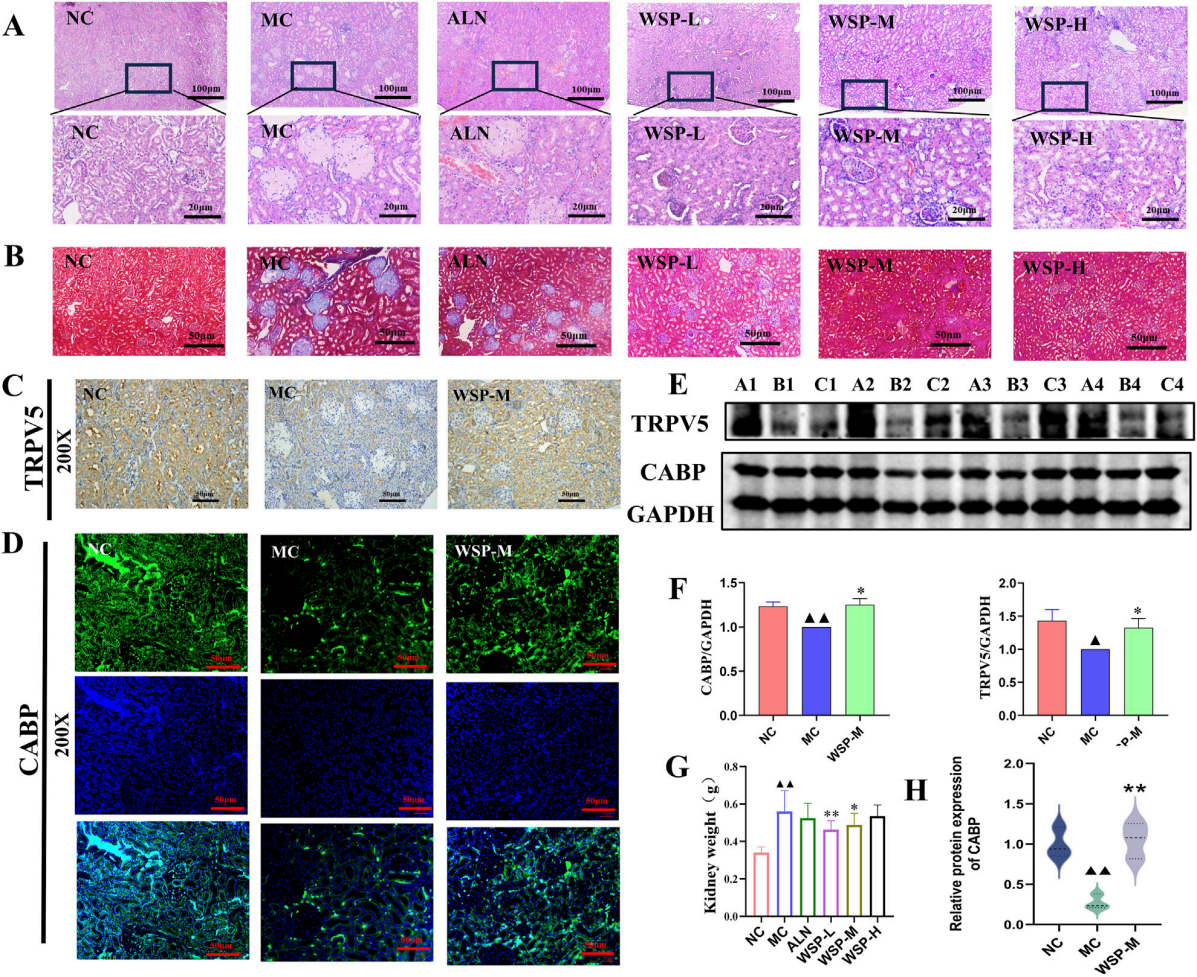
WSP increases renal calcium uptake in diet-induced PMOP mice. **(A)** H&E staining of mouse kidney (50x, 400x); **(B)** Masson staining of mouse kidney (200x); **(C)** Immunohistochemistry to detect the expression of TRPV5 in mouse kidney (200x); **(D)** Immunofluorescence to detect the expression of CABP in mouse kidney (200x); **(E,F)** Western blot to detect the expression of TRPV5 and CABP in kidney tissues (A1∼A4 for NC, B1∼B4 for MC, C1∼C4 for WSP-M group, n = 4); **(G)** weight of mouse kidney (n = 10). **(H)** Immunofluorescence-based relative quantification of CABP expression in the kidney. Data are presented as $\bar{x}$ ±s, ^▲▲^ *P* < 0.01, ^▲^ *P* < 0.05, compared with NC, **P < 0.01, *P < 0.05, compared with MC.

The regulation of calcium homeostasis in the kidney is primarily reflected through the reabsorption and excretion of calcium in the renal tubules. H&E and Masson staining showed that the kidney structure of NC mice was intact, with clearly defined glomerular and tubular structures, and no significant alterations in the cortical and medullary regions. Additionally, there was no aggregation of collagen fibers. In contrast, MC mice exhibited increased kidney volume, enlarged glomeruli, collagen fiber aggregation, inflammatory cell infiltration, and tubular dilation with cystic formation. However, in the WSP-treated groups, significant improvements in kidney histomorphology were observed, with noticeable restoration of normal kidney architecture compared to the MC group (Figures 6A,B). IHC and IF analysis revealed that the expression of TRPV5 and CABP-positive cells in the kidneys of MC mice was significantly reduced compared to both the NC and WSP-M groups (Figures 6C,D). Western blot analysis further confirmed that the expression of TRPV5 and CABP in the kidneys of MC mice was significantly lower (P < 0.01, 0.05), while WSP-M treatment led to a significant increase in TRPV5 and CABP expression in the kidneys compared to MC (P < 0.05) (Figures 6E,F). Additionally, the kidney wet weight of MC mice was significantly increased (P < 0.01), while the WSP-L group showed a significant decrease in kidney weight compared to the model group (P < 0.01, 0.05) (Figure 6G). Immunofluorescence quantification further confirmed the results shown in Figures C–F (Figure 6H) (P < 0.01). These findings suggest that WSP ameliorates renal injury and enhances calcium reabsorption in the kidneys of diet-induced PMOP mice.

### 3.7 WSP improves bone calcium absorption in diet-induced PMOP mice

**FIGURE 7:**
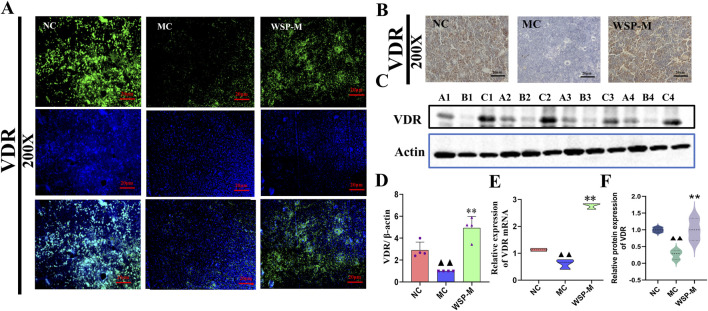
WSP promotes bone calcium deposition in diet-induced PMOP mice. **(A-D)** Expression of femoral VDR by immunofluorescence (400X), immunohistochemistry (400X), and Western blot; **(E)** Expression of femoral VDR mRNA by qRT-PCR; **(F)** Immunofluorescence-based relative quantification of VDR expression in the femur. Data are presented as $\bar{x}$ ±s, ^▲▲^ *P* < 0.01, ^▲^ *P* < 0.05, compared with NC, **P < 0.01, *P < 0.05, compared with MC.

The VDR serves as a nuclear receptor for 1,25-(OH)_2_D_3_, and its activation has been shown to attenuate the release of osteocalcin into the bloodstream. In both IF and IHC assays, we observed that the expression of VDR was significantly higher in the NC and WSP-M groups compared to the MC group (Figures 7A,B). Moreover, the results of Western blot and PCR analyses demonstrated that the expression of VDR at both the protein and mRNA levels was significantly (P < 0.01) lower in the femurs of the MC group mice compared to those in the NC group. In contrast, VDR protein and mRNA expression levels in the WSP-M group were significantly (P < 0.01) elevated when compared to the MC group (Figures 7C–E). Immunofluorescence quantification further confirmed the results shown in Figures A–E (Figure 7F) (P < 0.01). These findings indicate that the bone calcium absorption enhancement by WSP is associated with upregulation of VDR expression.

## 4 Discussion

Osteoporosis is categorized into two primary classifications: primary and secondary. Primary osteoporosis is largely a degenerative condition linked to the aging process, including postmenopausal, senile, and idiopathic osteoporosis (which primarily affects adolescents). Secondary osteoporosis is generally instigated by pharmacological interventions or other underlying medical conditions (Zhou et al., 2025). Calcium is a vital mineral in the human body, playing a crucial role in maintaining bone density. A reduction in calcium levels can lead to decreased bone density, ultimately contributing to the development of osteoporosis (Shen et al., 2022; Topolska et al., 2020; Wang and Li, 2022). The calcium intake and subsequent absorption are primarily allocated to skeletal mineralization, whereas unabsorbed calcium is primarily eliminated through fecal and urinary excretion. Consequently, calcium homeostasis is principally governed by the skeletal system, small intestine, and kidneys. During menopause, the reduction in estrogen levels precipitates osteoporosis, which is distinguished by heightened bone resorption, resulting in the mobilization of calcium and phosphorus from the skeletal matrix into the systemic circulation. This disruption of calcium balance leads to disorders in calcium metabolism. The regulation of calcium metabolism in the body involves several key factors, including PTH, CT, vitamin D_3_, and E_2_. PTH is a single-chain polypeptide hormone synthesized and secreted by the parathyroid glands. It plays a crucial role in regulating extracellular calcium and phosphorus levels. Elevated levels of PTH enhance bone resorption and increase calcium loss from the bones (Rejnmark and Ejlsmark-Svensson, 2020), thereby disrupting calcium homeostasis. CT primarily functions to inhibit the absorption of calcium ions in the small intestine and reduce calcium and phosphorus reabsorption in the distal renal tubules. Additionally, 1,25-(OH)_2_D_3_, the active form of vitamin D, regulates calcium absorption in the intestine. In postmenopausal women and during aging, estrogen deficiency can lead to relative insufficiency or resistance to active vitamin D, specifically 1,25(OH)_2_D_3_, which subsequently downregulates the expression of intestinal TRPV6 and renal TRPV5. This process ultimately impairs calcium absorption efficiency and elevates calcium loss. When intestinal calcium absorption is inadequate to maintain serum calcium levels, PTH secretion is upregulated. PTH enhances renal calcium reabsorption but primarily stimulates osteoclast-mediated bone resorption, resulting in the mobilization of bone calcium to stabilize serum calcium and promote bone loss (Hao et al., 2024). Physiologically, elevated serum calcium stimulates thyroid C cells to release CT, which counteracts PTH. CT significantly inhibits bone resorption by reducing osteoclast activity and proliferation, while also promoting renal calcium excretion; these actions collectively facilitate calcium deposition in bone, reduce serum calcium, and preserve bone integrity. In the postmenopausal state, in addition to the aforementioned calcium absorption deficits, estrogen deficiency may reduce basal CT secretion and attenuate its effects, thereby depriving the body of a critical endogenous mechanism to counteract bone resorption. This was additionally supported by increased serum concentrations of calcium and phosphorus (Luo et al., 2023).

The small intestine is recognized as the primary site for calcium absorption, with regional variations in absorptive capacity. The duodenum exhibits the highest calcium absorption rate, followed by the jejunum and ileum. (Fleet, 2022). These segments display an inverse relationship between absorption rate and retention time, with the ileum having the longest retention period, succeeded by the jejunum and duodenum (Bronner, 2009). An analysis of absorption efficiency and transit time indicates that the ileum and jejunum are the major sites for calcium absorption, accounting for approximately 65% and 17% of total intestinal calcium uptake, respectively (Christakos, 2012). The intestinal mucosa, particularly in the vicinity of the ileum, facilitates the absorption of calcium ions (Ca^2+^) through calcium transport channels associated with epithelial tight junction complexes. The only identified Ca^2+^-selective channels within this family are TRPV6 and TRPV5, which are members of the transient receptor potential (TRP) families (Clapham, 2003; Wang et al., 2025). TRPV6, a six-transmembrane domain epithelial calcium channel predominantly expressed in intestinal villi, plays a critical role in facilitating active calcium absorption by mediating Ca^2+^ influx from the intestinal lumen into enterocytes (Lieben et al., 2010). Simultaneously, CABP, characterized by its strong calcium affinity, is localized within enterocytes and the brush border epithelial cells of the small intestine. Following intracellular Ca^2+^ influx, CABP facilitates ion sequestration and promotes transcellular transport from the apical membrane to the basolateral compartment (Li et al., 2014).

Moreover, TRPV5 is predominantly situated in the distal and connecting tubules of the kidney, where it is instrumental in renal calcium reabsorption (Zhang et al., 2023). The kidney’s distal tubules facilitate calcium reabsorption via TRPV5, responsible for 15% of total calcium recycling; TRPV5 knockdown leads to elevated urine calcium, reduced blood calcium, and impaired bone mineralization (Luo et al., 2023). Renal calcium reabsorption is a tightly controlled process involving two main pathways: paracellular passive transport across the renal epithelium and transcellular active transport. The transcellular pathway occurs in three steps: first, calcium enters the epithelial cells through the apical membrane via TRPV5 channels; then, calcium binds to CABP and is transported across the cytoplasm; finally, calcium exits the cell via plasma membrane calcium ATPases (PMCA1 and PMCA4) and sodium-calcium exchanger isoform 1 (NCX1) (van Goor et al., 2017). A study on PMOP mice revealed substantial renal injury, characterized by increased renal volume, glomerular enlargement, collagen fiber aggregation, inflammatory cell infiltration, and tubular dilation into cystic structures. Moreover, the expression of calcium channel proteins, including TRPV5 and CABP, was markedly diminished. Ultimately, VDR serves as a nuclear receptor for 1,25-(OH)_2_D_3_, which, upon activation, enhances the absorption of osteocalcin and subsequently diminishes its secretion into the bloodstream (Benn et al., 2008; Gasperini et al., 2023). 1,25-(OH)_2_D_3_ can create a hormone-receptor complex with VDR, which subsequently interacts with additional regulatory variables to modulate VDR expression (Yang et al., 2015).

Previous studies have identified that the botanical components of WSP regulate pathways involved in bone metabolism, supporting its traditional use in strengthening kidney and spleen function (Han et al., 2021; Tang et al., 2023). However, the specific pharmacological mechanisms underlying the efficacy of WSP, as an integrated botanical compound, in the treatment of PMOP remain unclear. Current research indicates that numerous TCM compound prescriptions exert significant ameliorative effects on PMOP, with distinct mechanisms of action. Nevertheless, substantial gaps still exist in the understanding of their roles in regulating calcium-phosphorus absorption and bone metabolism (Liu et al., 2023). Distinct from most herbal interventions that primarily target anti-inflammatory pathways or exhibit estrogen-mimetic effects, WSP has demonstrated preliminary evidence of modulating calcium reabsorption in the intestine, kidney, and bone. In this study, we sought to develop a PMOP model by screening female mice exhibiting estrous cycle irregularities and subsequently administering a calcium-deficient, phosphorus-enriched diet. Assessment of general signs in these mice revealed clinical presentations consistent with human PMOP. These results validate the model’s capacity to replicate the features of primary menopausal osteoporosis observed in the current human population. Although the PMOP model established by combining natural menopause with dietary intervention avoids the invasiveness of ovariectomy, several limitations remain. The extreme calcium-phosphorus imbalance in the diet may overactivate PTH; meanwhile, individual variability in the extent of ovarian decline among mice contributes to phenotypic inconsistency and reduced reproducibility. Nevertheless, this model can still serve as a valuable complement to the ovariectomy model. Additionally, using 6-8-week-old mice as the normal control group introduces a confounding age effect. This cross-age experimental design results in differences in baseline bone metabolism, endocrine status, and physiological function between groups, which is also an issue that should be considered in subsequent studies.

In conclusion, our data indicate that WSP mitigates osteoporotic alterations in a diet-induced murine model by augmenting calcium absorption via the gut-kidney-bone axis. These results offer initial mechanistic understanding of calcium regulation in PMOP, while validation in clinical trials is warranted.

## 5 Conclusion

**FIGURE 8:**
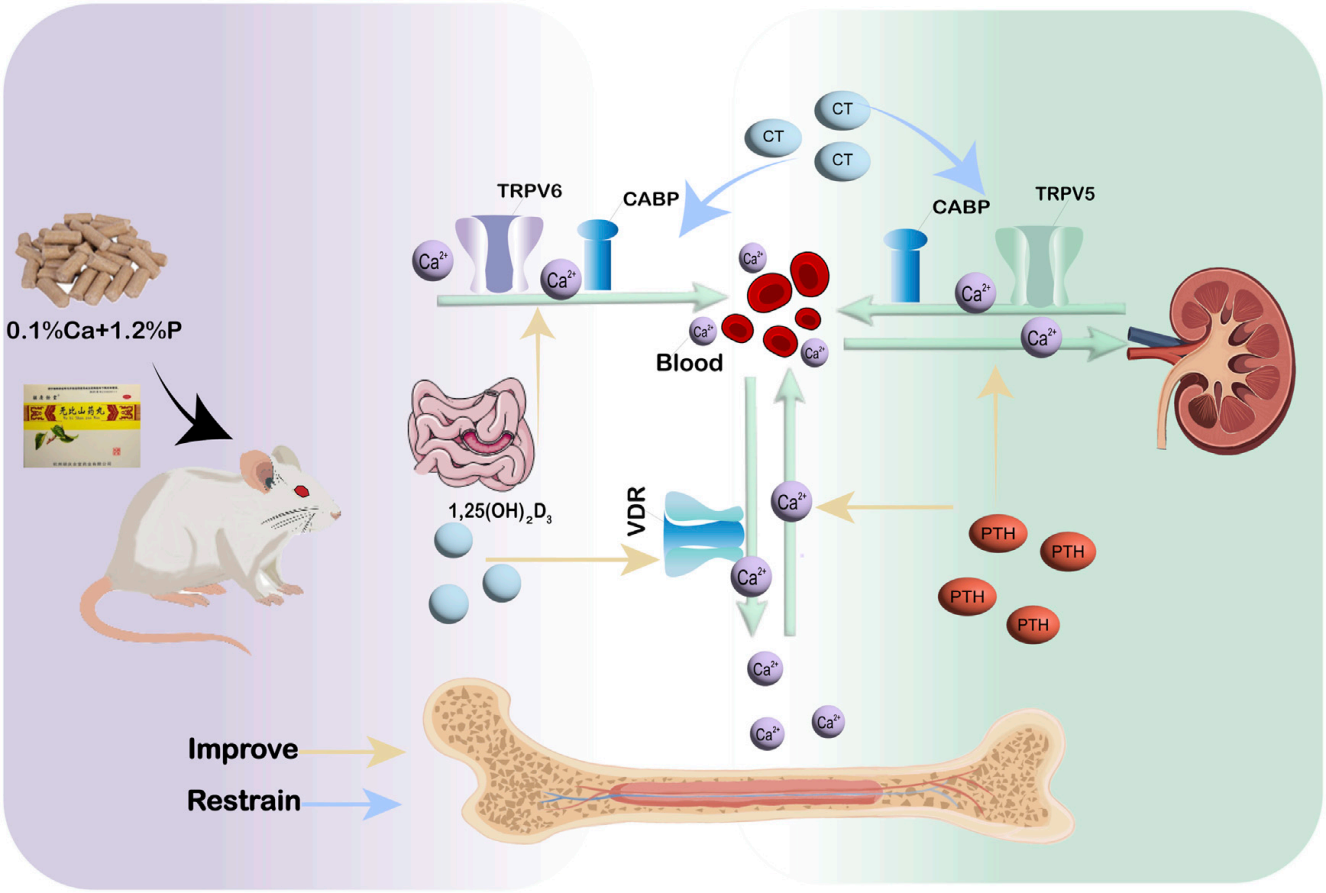
WSP’s Calcium Absorption Mechanism in diet-induced PMOP mice.

In summary, this study reveals that WSP treatment mitigates osteoporotic manifestations in a diet-induced murine model of PMOP. The observed therapeutic effects are associated with augmented calcium absorption, substantiated by the upregulation of critical calcium transport proteins (TRPV5/6, CABP, and VDR) within the gut–kidney–bone axis. Concurrently, WSP improved bone mass, restored calcium–phosphorus homeostasis, ameliorated systemic metabolism, and reversed anemia in this model. These observations suggest that WSP facilitates structural repair in the ileum, kidney, and femur, thereby integrating multi-tissue regeneration with calcium regulatory pathways (Figure 8). Collectively, these preclinical findings offer mechanistic insights into the role of calcium absorption in PMOP pathophysiology and support further exploration of WSP as a potential therapeutic intervention. However, given that the current evidence is limited to a diet-induced mouse model, additional studies are warranted to validate its translational applicability in human PMOP.

## Data Availability

The raw data supporting the conclusions of this article will be made available by the authors, without undue reservation.
